# Biocompatibility and Biocorrosion of Hydroxyapatite-Coated Magnesium Plate: Animal Experiment

**DOI:** 10.3390/ma10101149

**Published:** 2017-09-30

**Authors:** Ho-Kyung Lim, Soo-Hwan Byun, Jae-Man Woo, Sae-Mi Kim, Sung-Mi Lee, Bong-Ju Kim, Hyoun-Ee Kim, Jung-Woo Lee, Soung-Min Kim, Jong-Ho Lee

**Affiliations:** 1 Department of Oral and Maxillofacial Surgery, Korea University Medical Center, Guro Hospital, Seoul 08308, Korea; ungassi@naver.com; 2 Department of Oral and Maxillofacial Surgery, Dongtan Sacred Heart Hospital, Hallym University Medical Center, Kyonggi-do 18450, Korea; purheit@hanmail.net; 3 Department of Oral and Maxillofacial Surgery, Seoul National University Dental Hospital, Seoul 03080, Korea; jmanwoo@gmail.com (J.-M.W.); smin5@snu.ac.kr (S.-M.K.); 4 Department of Material Science and Engineering, Seoul National University, Seoul 08826, Korea; bluelear@snu.ac.kr (S.-M.K.); msbb419@snu.ac.kr (S.-M.L.); kimhe@snu.ac.kr (H.-E.K.); 5 Clinical Translational Research Center for Dental Science, Seoul National University Dental Hospital, Seoul 03080, Korea; bjkim016@gmail.com; 6 Department of Oral and Maxillofacial Surgery, Kyunghee University Dental Hospital, Seoul 02453, Korea

**Keywords:** magnesium, hydroxyapatite, biocompatibility, biocorrosion

## Abstract

Magnesium (Mg) has the advantage of being resorbed in vivo, but its resorption rate is difficult to control. With uncontrolled resorption, Magnesium as a bone fixation material has minimal clinical value. During resorption not only is the strength rapidly weakened, but rapid formation of metabolite also occurs. In order to overcome these disadvantages, hydroxyapatite (HA) surface coating of pure magnesium plate was attempted in this study. Magnesium plates were inserted above the frontal bone of Sprague-Dawley rats in both the control group (Bare-Mg group) and the experimental group (HA-Mg group). The presence of inflammation, infection, hydrogen gas formation, wound dehiscence, and/or plate exposure was observed, blood tests were performed, and the resorption rate and tensile strength of the retrieved metal plates were measured. The HA-Mg group showed no gas formation or plate exposure until week 12. However, the Bare-Mg group showed consistent gas formation and plate exposure beginning in week 2. WBC (White Blood Cell), BUN (Blood Urea Nitrogen), Creatinine, and serum magnesium concentration levels were within normal range in both groups. AST (Aspartate Aminotransferase) and ALT (Alanine Aminotransferase) values, however, were above normal range in some animals of both groups. The HA-Mg group showed statistically significant advantage in resistance to degradation compared to the Bare-Mg group in weeks 2, 4, 6, 8, and 12. Degradation of HA-Mg plates proceeded after week 12. Coating magnesium plates with hydroxyapatite may be a viable method to maintain their strength long enough to allow bony healing and to control the resorption rate during the initial period.

## 1. Introduction

Plates and screws made of various materials have been used to facilitate bone healing in the oral and maxillofacial region such as in fracture reduction and orthognathic surgery. Plates and screws made of titanium (Ti) alloy are widely used because of their excellent mechanical strength. However, titanium alloy is non-resorbable, releases trace amount of harmful materials that may have an effect in body fluid [1], increases risk of infection when they are covered with non-vascular fibrous tissue [1], and causes the distortion of CT (Computed Tomography) and MRI (Magnetic Resonance Imaging). There is also risk of inhibiting growth and development at the surgical site in growing children [2]. For these reasons, titanium alloy plates and screws often require removal which entails additional surgery, resulting in repeated surgical trauma and financial burden to the patient.

Currently available resorbable fixation systems include those made of polylactic acid (PLA), polyglycolic acid (PGA), and their polymer, Poly l-lactide-*co*-glycolide (PLGA). These materials have lower strength, larger size and volume, and difficult handling characteristics compared to metal alloys [2]. Also, resorption of these materials occurs over a long period of time [3] and the pH of surrounding tissue tends to decrease during degradation, causing inflammation and necrosis of the surrounding tissue [4].

Magnesium (Mg) as a mineral is crucial to the body’s various functions, while magnesium as a metal is a non-toxic biodegradable material that has higher strength and excellent handling characteristics compared to its polymeric counterparts [5]. In addition, magnesium has a modulus of elasticity comparable to human bone, which may prevent stress shielding phenomenon, a common problem of metal and metal alloy fixation systems [6]. Magnesium, however, undergoes rapid corrosion in certain environments such as when in contact with body fluid where chloride ions cause the early deterioration of strength and hydrogen gas formation. During the corrosive degradation, the pH of the area increases significantly, which may lead to inflammatory reaction of the surrounding tissue [7].

Mg + 2H_2_O→Mg^2+^ + 2OH^−^ + H_2_

Various methods have been devised and developed to overcome the rapid corrosion of magnesium, and one widely-known method is surface modification [8]. In this study, magnesium plates were coated with hydroxyapatite (HA) to maintain mechanical strength, increase biocompatibility, and control biodegradation rate in body fluid. The prepared plate specimens were tested in vivo in order to study local inflammatory reaction, physiologic response, degradation rate, and the degree of maintenance of tensile strength.

## 2. Materials and Methods

### 2.1. Specimen Preparation

**Figure 1:**
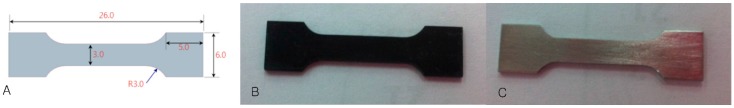
Magnesium (Mg) plate specimens. (**A**) Design of the magnesium plates; (**B**) Hydroxyapatite-coated magnesium plate (HA-Mg group); (**C**) Uncoated magnesium plate (Bare-Mg group).

High-purity (99.99%) magnesium was intensified via the bi-axial rolling process to enhance mechanical strength. Magnesium plates were designed (*n* = 55, 26 × 6 × 2 mm^3^) for strength measurement (Figure 1), mechanically polished using 1200 grit SiC paper, washed in an ultrasonic water bath, and dried. The method for the hydroxyapatite coating process proposed by Hiromoto et al. [9] was followed: specimens were soaked in 0.5 M ethylenediaminetetraacetic acid calcium disodium salt hydrate (Ca-EDTA) and 0.05 M potassium dihydrogenphosphate (KH_2_PO_4_) solution, then heat-processed at 363 K for 2 h. The pH was maintained at 8.9 by adding sodium hydroxide (NaOH) solution. The HA-coated specimens were then washed with distilled water and dried.

### 2.2. Animal Surgery

**Figure 2:**
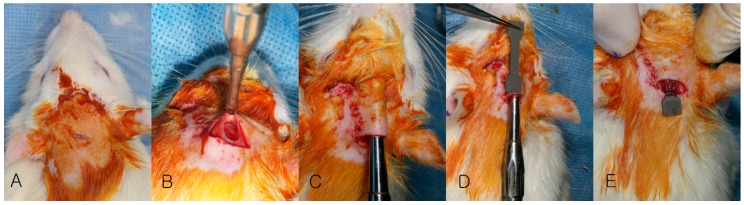
Surgical procedure of plate insertion. (**A**) Shaving of hair; (**B**) Incision and dissection; (**C**) Sub-periosteal envelope formation; (**D**) Measuring of envelop size; (**E**) Insertion of magnesium plate.

Fifty-five 10-week-old Sprague-Dawley rats (body weight of 300–350 g, Orient Corp., Seoul, Korea) were randomly divided into two groups after a two-week adaptation period. Uncoated magnesium plates were inserted into the calvaria of 25 rats (Bare-Mg group), and hydroxyapatite-coated magnesium plates were inserted into the calvaria of 30 rats (HA-Mg group). Rats were put under general anesthesia using a mixture of xylazine (Rompun^®^, 20 mg/mL, Bayer Korea Ltd., Seoul, Korea) and ketamine hydrochloride (Ketalar^®^, 50 mg/mL, Yuhan Corp., Seoul, Korea) (1:4 volumes, dosage 0.15 mL/100 g) delivered via intraperitoneal injection. The cranial hair was shaved, and the exposed skin was sterilized by povidone-iodine painting. Transverse skin incision was made and the periosteum was elevated with a periosteal elevator. The Mg plate was inserted carefully underneath the periosteum and the flap was closed using 5-0 nylon (Ethicon^®^, Johnson & Johnson, New Brunswick, NJ, USA) (Figure 2). Five rats each were sacrificed using CO_2_ asphyxiation at 2, 4, 6, 8, and 12 weeks in the Bare-Mg group, and 2, 4, 6, 8, 12, and 24 weeks in the HA-Mg group.

### 2.3. Evaluation

#### 2.3.1. Clinical Evaluation

Clinical findings such as inflammation, infection, gas formation, wound dehiscence, and plate exposure were recorded at the time of sacrifice. Magnesium plates were retrieved and the absorption pattern of each plate was examined.

#### 2.3.2. Hematological Evaluation

In order to establish baseline values of blood test results, blood samples were taken from three healthy rats not included in the study. Blood samples were collected before the rats were sacrificed. White blood cell (WBC), blood magnesium (Mg^2+^), alanine aminotransferase (ALT) and aspartate aminotransferase (AST) for liver function assessment, and blood urea nitrogen (BUN) and serum creatinine (Cr) for kidney function assessment were measured.

#### 2.3.3. Evaluation of Absorption Rate using µCT

Micro CT (µCT) (Skyscan 1172 Micro-tomography System, Skyscan, Kontich, Belgium) scan of Mg plates were done before insertion and at sacrifice to examine the absorption rate and pattern. Volume of the plates was constructed from the sectioned images using the Feldkamp algorithm. The computation for the absorption rate (DRi) is shown below.

$$\mathrm{DRi}=\frac{\mathrm{Vi}}{V}\times 100\%,\;\mathrm{Vi}=V-\mathrm{Vci}$$

Sectional images of the plates were rendered three-dimensionally (V) using the CTvox program (Skyscan, Kontich, Belgium) prior to insertion. The volume at the time of sacrifice (Vi) is the volume of the plate before the experiment (V) minus the measured volume of the constructed three-dimensional absorbed area (Vci).

#### 2.3.4. Change of Mechanical Strength

Mg plates were retrieved after µCT. The tensile strength of the retrieved plates was measured using the universal testing machine (OTU-05D, Oriental TM Corp., Seoul, Korea) with a crosshead speed of 1 mm/min.

#### 2.3.5. Statistical Analysis

Results of the blood test, degradation rate, and tensile strength were analyzed by Student’s *t*-test, two-way analysis of variance (ANOVA), and repeated measurement ANOVA, and confirmed with post-hoc Bonferroni’s multiple comparison test (*p* < 0.05) using the SPSS 22.0 program (IBM SPSS Statistics, IBM Corp., Somers, NY, USA).

## 3. Results

### 3.1. Clinical Evaluation

**Figure 3:**
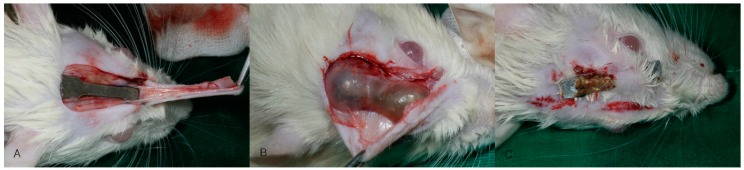
Clinical findings at sacrifice. (**A**) Normal; (**B**) Hydrogen gas formation; (**C**) Wound dehiscence and plate exposure.

All rats recovered immediately after surgery. Clinical examination showed no specific findings up to one week postoperatively. In the Bare-Mg group, gas formation and plate exposure were observed starting at week 2 of the experiment. In the HA-Mg group, gas formation was not observed until week 12. Gas formation was observed at 24 weeks, but plate exposure was not observed during the whole period in the HA-Mg group (Figure 3 and Table 1).

**Figure 4:**
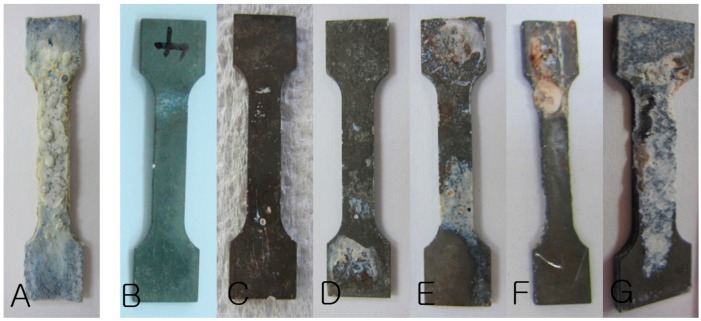
Retrieved magnesium plates: (**A**) Week 2 specimen of the Bare-Mg group, (**B**–**G**) Weeks 2, 4, 6, 8, 12, and 24 specimens of the HA-Mg group.

Corrosion products covered the surfaces of Bare-Mg group plates starting at week 2. The HA-coated Mg plates showed no significant change until week 2, but the HA-coating started to strip off in pin-point-like fashion at week 4 and gradually progressed until week 12. Degradation of magnesium was observed on surfaces stripped of the HA coating (Figure 4).

### 3.2. Hematological Evaluation

**Figure 5:**
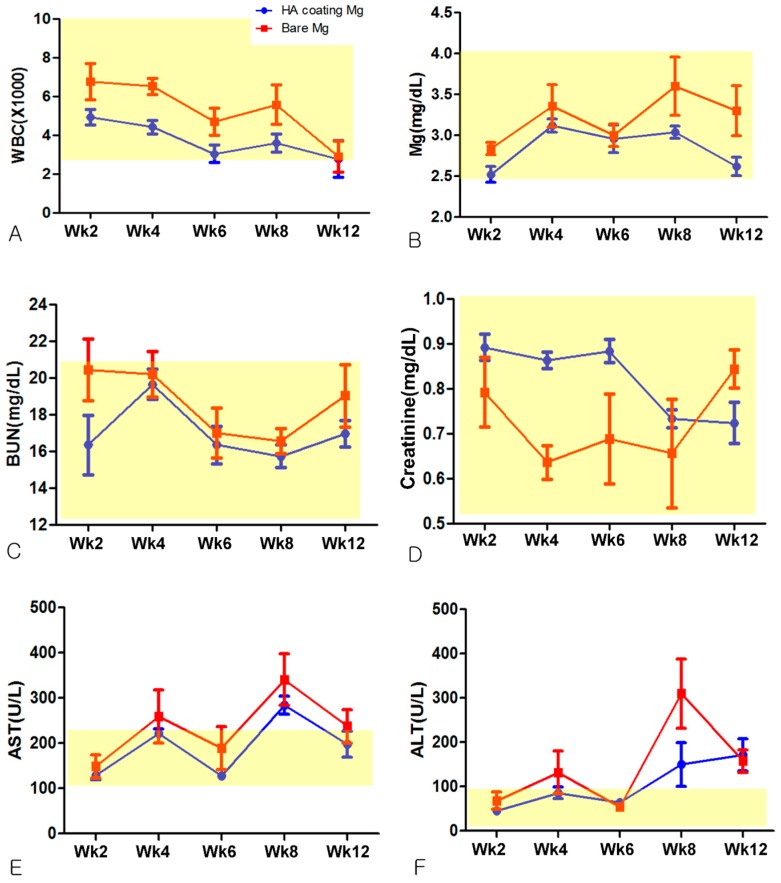
Hematological findings (normal range highlighted in yellow). (**A**) White Blood Cell count (WBC); (**B**) Blood Magnesium level (Mg^2+^); (**C**) Blood urea nitrogen (BUN); (**D**) Serum creatinine; (**E**) Aspartate aminotransferase (AST); (**F**) Alanine aminotransferase (ALT).

WBC, BUN, Creatinine, and blood magnesium levels were within normal range in both groups. Lab values were generally higher in the Bare-Mg group. AST and ALT values were higher than norm in some rats in both groups. The Bare-Mg group showed higher average AST and ALT value compared to the HA-Mg group (Figure 5).

### 3.3. Evaluation of Absorption Rate Using µCT

**Figure 6:**
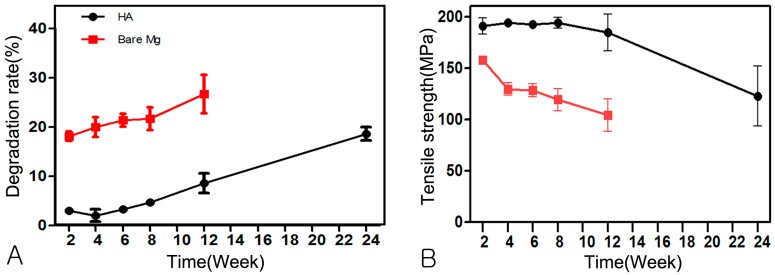
Degradation rate and tensile strength: (**A**) Degradation rate; (**B**) Tensile strength.

**Figure 7:**
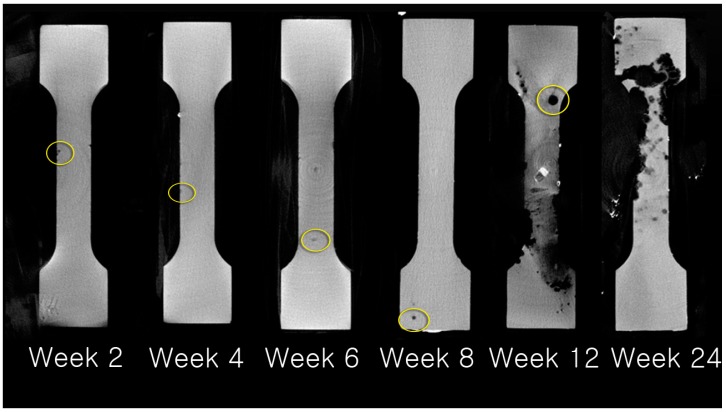
Pattern of resorption of HA-Mg plates examined using micro-computed tomography. Resorption had pin-point-like appearance until week 8 (yellow circles). Resorption progressed rapidly beginning at week 12.

The HA-Mg group showed statistically significant (*p* < 0.001) advantage in resistance to absorption, demonstrating slower absorption than the Bare-Mg group. Active absorption of HA-coated Mg plates was not observed until week 12 (Figure 6 and Figure 7, and Table 2).

### 3.4. Change of Mechanical Strength

HA-coated Mg plates maintained tensile strength for 12 weeks (>190 MPa). Bare Mg plates, however, quickly lost tensile strength beginning at week 2 after insertion (*p* < 0.05), and the strength continuously decreased significantly with time (*p* < 0.05). The tensile strength of the plates did not exceed 200 MPa (Figure 6 and Table 3).

## 4. Discussion

While magnesium has many advantages such as resorbability, easy handling characteristics, and adequate strength, one major disadvantage is its high in vivo corrosion rate. Corrosion results in the rapid deterioration of physical properties and the release of corrosion byproducts such as hydrogen gas. One way to overcome this problem is surface modification by HA coating. There are several methods of HA coating such as sol gel [10], aerosol deposition [11], electrophoretic deposition [12], electron beam deposition [13], and pulsed laser deposition [14]. This study followed the coating method proposed by Hiromoto et al. [9]. Following this method, Kim et al. [8] coated the bare magnesium surface with a uniform HA layer with a thickness of 2 μm, obtained 20 MPa binding force between the magnesium surface and the HA layer, and confirmed the decrease of gas formation, acidification, and magnesium metal debris in an in vitro experiment. Furthermore, the surface cell proliferative capacity was also improved.

Positive effects of HA coating were noted in this study as well. In the Bare-Mg group, gas formation and plate exposure was observed consistently throughout the study starting at week 2, while the HA-Mg group showed no gas formation or plate exposure until week 12. Retrieved Bare-Mg plates were covered with corrosion byproducts starting at week 2, while HA-Mg plates showed a more localized and gradual surface change. The coating of the HA-Mg plates started to strip off with a pin-point like appearance and corrosion byproducts gradually developed on the exposed magnesium surface (Figure 7). The HA coating layer seems to deteriorate rapidly after week 12 and lose its anti-corrosive effect. Such a difference in clinical findings between the two groups indicates that (1) the corrosion process begins as soon as there is exposure of bare magnesium to the external environment and (2) the HA coating allows a more gradual and localized surface corrosion at least for the initial 12-week period.

Lim et al. [15], in 2016, reported that the blood magnesium level was found to be within the normal range when WE43 Mg alloy was inserted on the tibia of rabbits. Zhang et al. [16] ran blood tests after inserting a Mg-Zn-Mn alloy on the femur of rats, and the result showed elevated BUN, serum creatinine, AST, and ALT above the norm. They theorized that resorbed magnesium may be metabolized in the kidneys. The results of this study, however, showed blood lab results within normal limits except for AST and ALT, which are indicators of liver function. Although the lab results showed generally higher mean values in the Bare-Mg group except for serum creatinine on week 12, no statistically significant difference between groups was noted except for higher WBC level in the Bare-Mg group at week 4 (*p* < 0.05). These results show that in vivo corrosion and absorption of magnesium does not affect hematologic functions significantly. The elevation of AST and ALT values is presumably due to post-operative stress or the insertion of a large-volume magnesium plate relative to the size of the rat.

There has been a number of studies on the in vivo resorption rate of magnesium. Fischerauer et al. [17] compared the resorption of ZX50, a type of magnesium alloy, and micro-arc oxidation surface-treated ZX50 inserted in the femur of rats. The results showed that the resorption of untreated ZX50 started at week 1 and continued until week 12, and that of surface-treated ZX50 started at week 2 until week 8. In the study by Gu et al. [18], resorption was observed from post-operative week 2 when magnesium-strontium implants were inserted in the medullary bone of rats. In the study by Dziuba et al. [19], the volume of the ZEK100 magnesium alloy implants inserted in New Zealand white rabbits started to decrease at week 20. Considering the bone healing time following orthognathic surgery or fracture surgery, fixation plates should be able to retain physical properties and resist early resorption until post-operative week 4 at least, and the initiation of resorption is desirable after post-operative week 6 or 8 [20]. The results of this study showed that the HA-Mg plates maintained their volume and strength until week 12. Resorption was observed with a decrease in strength after week 12. These results shows that HA-Mg plates have the physical and resorption properties required for adequate bone healing.

## 5. Conclusions

HA-coated magnesium plates inserted in rat calvaria resisted resorption and weakening of tensile strength until week 12. The delayed and gradual resorption of HA-coated plates also showed reduced clinically observable side effects such as the rapid release of the hydrogen gas, wound dehiscence, or plate exposure. The results of this study indicate that magnesium plates with HA coating are biocompatible and maintain sufficient tensile strength for a period long enough to allow adequate bone healing. It also seems that the initial resorption rate of the magnesium plates can be controlled by HA coating.

## Figures and Tables

**Table 1:** Clinical findings at sacrifice.

Time	Hydrogen Gas Formation		Plate Exposure	
	HA-Mg	Bare-Mg	HA-Mg	Bare-Mg
Week 2	0	4	0	4
Week 4	0	3	0	2
Week 6	0	2	0	3
Week 8	0	4	0	1
Week 12	0	4	0	3
Week 24	2	-	0	-

HA-Mg: HA-coated magnesium plate group, Bare-Mg: untreated magnesium plate group, Unit: number of animals.

**Table 2:** The resorption rate (%) of the HA-Mg group and Bare-Mg group using micro-computed tomography.

Time	Week 2	Week 4	Week 6	Week 8	Week 12	Week 24
HA-Mg	2.99 ± 0.59	2.01 ± 2.77	3.33 ± 1.25	4.71 ± 1.49	8.58 ± 4.57	18.35 ± 3.03
Bare-Mg	18.10 ± 2.12 *	20.00 ± 4.47 *	21.40 ± 2.96 *	21.71 ± 5.20 *	26.70 ± 8.72 *	-

Values presented as mean values of all samples of one time interval ± standard deviation. * Significant difference (*p* < 0.05) between the HA-Mg group and Bare-Mg group.

**Table 3:** The results of the tensile strength (MPa) measurement.

Time	Week 2	Week 4	Week 6	Week 8	Week 12	Week 24
HA-Mg	190.10 ± 17.46	193.89 ± 5.19	192.16 ± 7.82	193.77 ± 11.77	184.58 ± 35.69	122.78 ± 65.07
Bare-Mg	157.94 ± 6.18 *	129.65 ± 13.70 *	128.40 ± 14.58 *	119.39 ± 24.15 *	104.22 ± 31.17 *	-

Values presented as the means of all samples of one time interval ± standard deviation. * Significant differences (*p* < 0.05) between the HA-Mg group and Bare-Mg group.
